# Spongiatriol Inhibits Nuclear Factor Kappa B Activation and Induces Apoptosis in Pancreatic Cancer Cells

**DOI:** 10.3390/md11041140

**Published:** 2013-04-02

**Authors:** Esther Guzmán, Michael Maher, Alexis Temkin, Tara Pitts, Amy Wright

**Affiliations:** Center for Marine Biomedical and Biotechnology Research, Harbor Branch Oceanographic Institute at Florida Atlantic University, 5600 US 1 North, Fort Pierce, FL 34946, USA; E-Mails: mmaher@umn.edu (M.M.); alexis.temkin@gmail.com (A.T.); tpitts3@hboi.fau.edu (T.P.); awrigh33@hboi.fau.edu (A.W.)

**Keywords:** inflammation, pancreatic cancer, nuclear factor kappa B, apoptosis

## Abstract

Pancreatic cancer, the fourth leading cause of cancer death in the US, is highly resistant to all current chemotherapies, and its growth is facilitated by chronic inflammation. The majority of pro-inflammatory cytokines initiate signaling cascades that converge at the activation of the Nuclear Factor Kappa B (NFκB), a signal transduction molecule that promotes cell survival, proliferation and angiogenesis. In an effort to identify novel inhibitors of NFκB, the HBOI library of pure compounds was screened using a reporter cell line that produces luciferin under the transcriptional control of NFκB. Seven compounds were identified through this screen, but in the case of five of them, their reported mechanism of action made them unlikely to be specific NFκB inhibitors. Spongiatriol, a marine furanoditerpenoid that was first isolated in the 1970s, is shown here to inhibit NFκB transcriptional activity in a reporter cell line, to reduce levels of phosphorylated (active) NFκB in the AsPC-1 cell line, to have an IC_50_ for cytotoxicity in the low micromolar range against the AsPC-1, BxPC-3, MiaPaCa-2 and Panc-1 pancreatic cancer cell lines, and to induce moderate but significant apoptosis in both the AsPC-1 and the Panc-1 cell lines.

## 1. Introduction

Pancreatic cancer is the fourth leading cause of cancer death in the United States. More than 44,000 people are expected to be diagnosed with the disease in 2013 and of those, only 26% will survive one year post diagnosis and only 6% will survive five years post diagnosis [1]. These statistics highlight the need for new treatments for this disease. 

Adult cancers are frequently preceded by chronic inflammation [2,3,4]. Chronic inflammatory diseases that are associated with malignancies include asbestosis and bronchitis with lung cancer, gingivitis with oral squamous cell carcinoma, colitis with colon cancer, skin inflammation with melanoma and pancreatitis with pancreatic cancer [2,4] Pancreatic ductal adenocarcinoma is highly resistant to all current chemotherapies, and its growth is often facilitated by chronic inflammation [5,6,7].

The majority of pro-inflammatory cytokines initiate signaling cascades that converge at the activation of the Nuclear Factor Kappa B (NFκB), a signal transducer, which promotes cell survival, proliferation and angiogenesis [8]. In pancreatic cancer and pancreatic cancer cell lines, NFκB is often constitutively activated [9] and its activation correlates with metastatic potential [10]. In an effort to discover small molecule inhibitors of NFκB from our library of marine natural products, ninety-two compounds from our pure natural products library were screened using a luciferase reporter cell line that contains luciferin under transcriptional control of NFκB. While we found various known compounds with this activity, their response could be ascribed to their previously described mode of actions as protein synthesis inhibitors, DNA intercalators or inhibitors of topoisomerase II. Spongiatriol, a marine furanoditerpenoid that was first isolated in the 1970s [11], was found to be active in the assays and did not have a reported mechanism of action that explained the inhibition of NFκB. Therefore, we undertook studies to better understand its effects in pancreatic cancer cell lines.

## 2. Results and Discussion

To assay for inhibitors of NFκB, a commercially available reporter cell line with luciferin under the transcriptional control of NFκB (A549 NFκB-luc) was used to screen the HBOI pure compound library. While this reporter cell line is not a pancreatic cancer cell line, it was chosen as it had been validated by other researchers, was commercially available and it could test for inhibition of transcriptional activity of NFκB. The assay is run by treating the A549 NFκB-luc cells with 100 ng/mL tumor necrosis factor alpha (TNF-α) to stimulate NFκB activity. Cells are then treated with either vehicle control or marine compounds. Compounds with no effect on NFκB produce luciferin which upon treatment with luciferase produces visible light which can be read as luminescence on a plate reader. Those that inhibit NFκB show reduced light production. Ninety-two compounds from the HBOI pure compound library were tested in this study. A549 NFκB-luc cells were treated with TNFα in the presence or absence of 5 μg/mL compounds or their respective solvent controls for six hours. Those that caused more than 50% inhibition of luminescence versus vehicle control with less than 20% cytotoxicity were selected as hits. Table 1 shows the average inhibition of lead compounds in three experiments ± standard deviation.

**Table 1:** Hits from screening the HBOI pure compound library.

Name	Structure class	Moleculer weight	Percent inhibition 5 μg/mL
Discalamide-A	Complex polyketide	623	97.2 ± 0.5
Discorhabdin-A	Beta carboline alkaloid	415	92.1 ± 2.8
Isobatzelline C	Alkaloid	235	98.8 ± 0.5
Mycalamide-B	Complex polyketide	517	97.0 ± 0.3
Onnamide-A	Complex polyketide	793	96.4 ± 1.0
Secobatzelline A	Alkaloid	255	99.1 ± 0.2
Spongiatriol	Terpene	364	91.0 ± 1.2

**Figure 1:**
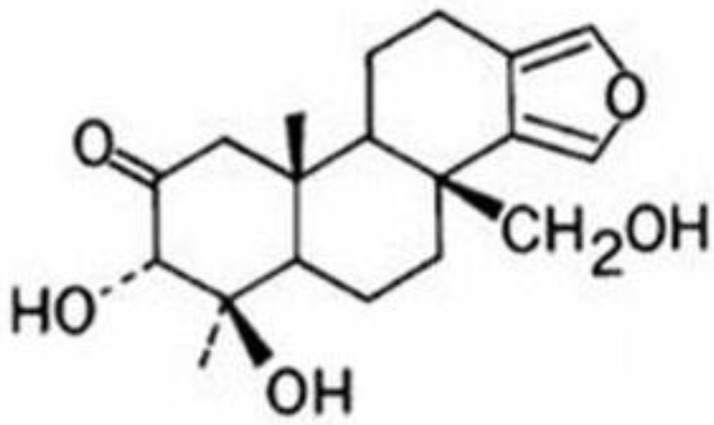
Structure of spongiatriol.

Onnamide A, mycalamide B and discalamide A are structurally related compounds. Onnamide and mycalamide B have been reported to inhibit protein synthesis and thus would be predicted to show activity in the reporter assay [12]. Discalamide A is related to these compounds and to the theopederins, the latter of which have also been reported to inhibit protein synthesis [13]. Isobatzelline C intercalates into DNA and secobatzelline A inhibits topoisomerase II activity [14]; both of these activities would lead to transcriptional inhibition. Therefore, of the actives from the primary assay, spongiatriol and discorhabdin A may show specific inhibition of NFκB and warrant further investigation. Spongiatriol was selected to validate this activity. Figure 1 shows its structure.

**Figure 2:**
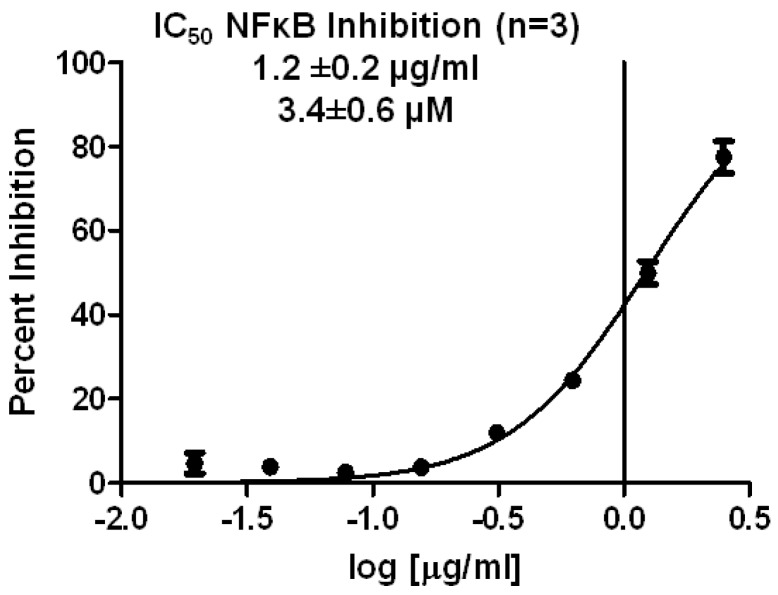
Non-linear regression curve for inhibition of NFκB transcriptional activity obtained from serial dilutions of spongiatriol. The graph represents the average of 3 experiments. The values shown are the average of 3 experiments ± standard deviation.

The first experiment performed was to determine the concentration needed to cause 50% inhibition of NFκB transcriptional activity (IC_50_) in the reporter cell line. As shown in Figure 2, spongiatriol had an IC_50_ of 3.4 ± 0.6 μM, which was calculated by using a non-linear regression curve fit with Graph Pad software. 

Because a major focus in our laboratory is to find compounds with the potential to be useful in the treatment of pancreatic cancer, we studied the effects of spongiatriol in pancreatic cancer cells known to express constitutively active NFκB [9]. In the reporter cell line, NFκB activity was induced through stimulation with 100 ng/mL TNFα while in the pancreatic cancer cells no further stimulation was required due to the constitutive activation of NFκB in these cells. To maximize the opportunity of observing the effects in the pancreatic cancer cell lines, spongiatriol was tested at two times the IC_50_ found for the reporter cell line (6.8 μM or 2.4 μg/mL). Inhibition of NFκB and effects on downstream signaling was measured at 6 h to replicate the timing used in the screening assay. Cytotoxicity was measured in the pancreatic cancer cell lines at 72 h using a standard MTT-based protocol. 

**Figure 3:**
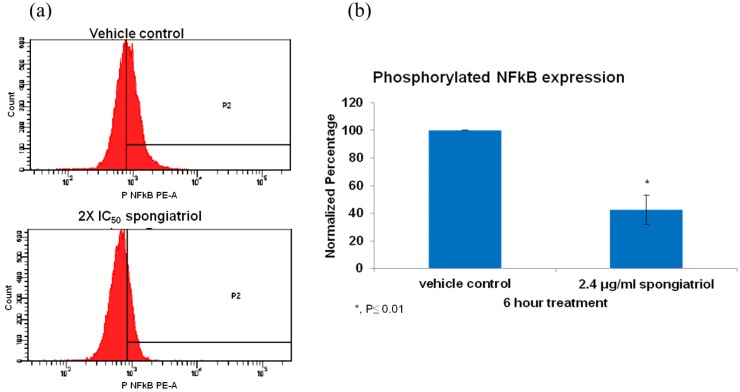
(**a**) Flow cytometry histograms for phosphorylated NFκB p65 (pS529) in AsPC-1 cells treated for 6 h with vehicle control or 6.8 μM (2.4 μg/mL) spongiatriol (2× IC_50_ for NFκB inhibition in reporter cell line). Gates were set up against isotype control. One representative experiment is shown. (**b**) Graphical representations of the flow cytometry data normalized to vehicle control showing the average of three experiments. Error bars represent standard deviation. Statistical significance was determined through the Student’s *t*-test.

The pancreatic adenocarcinoma cell line AsPC-1, has constitutively activated NFκB and is highly metastatic and resistant to apoptosis and was used to investigate whether spongiatriol could reduce levels of activated NFκB in a highly metastatic cell line. Flow cytometry measuring the levels of phosphorylated (active) NFκB p65 (pS529) with respect to those observed in vehicle control indicated that spongiatriol significantly reduced the levels of NFκB p65 (pS529) in AsPC-1 cells (Figure 3).

While the compounds chosen exhibited little or no cytotoxicity in the reporter cell line during the 6 h treatment time (which was of importance to eliminate false positives), we expected that the compounds would have a different effect in cells that express constitutively active NFκB. NFκB regulates over 500 genes (elegantly reviewed by Gupta *et al.* [15]). Particularly relevant for pancreatic cancer cells is the regulation of anti-apoptotic proteins, regulation of cell cycle progression and cytokine production [15] which may contribute to the resistance to apoptosis and high metastatic potential exhibited by many pancreatic cancer cells [9,10,16]. NFκB is a known regulator of anti-apoptotic molecules and the inhibition of constitutively activated NFκB has been shown to sensitize cells to apoptosis in pancreatic cancer cells [16]. Therefore, we expected that inhibition of the constitutively activated NFκB in the pancreatic cancer cell lines would result in induction of apoptosis. The cytotoxicity of spongiatriol in four pancreatic cancer cell lines was determined using a standard MTT-based protocol. As shown in Table 2, spongiatriol induced cytotoxicity in these cell lines in the low micromolar range.

**Table 2:** Concentration of spongiatriol needed to obtain 50% cytotoxicity (IC_50_) in four pancreatic cancer cell lines. Cells were incubated for 72 h in the presence of serial dilutions of spongiatriol. Proliferation was determined by following the reduction of the tetrazolium salt 3-(4,5-dimethylthiazol-2-yl)-2,5-diphenyltetrazolium bromide (MTT) into a formazan, normalized to solvent control and subjected to a non-linear regression analysis. Data is the average of three experiments ± standard deviation.

Cell lines	IC_50_ cytotoxicity (μM)
AsPC-1	13 ± 2
BxPC-3	8 ± 3
MiaPaCa-2	6 ± 1
Panc-1	13 ± 5

**Figure 4:**
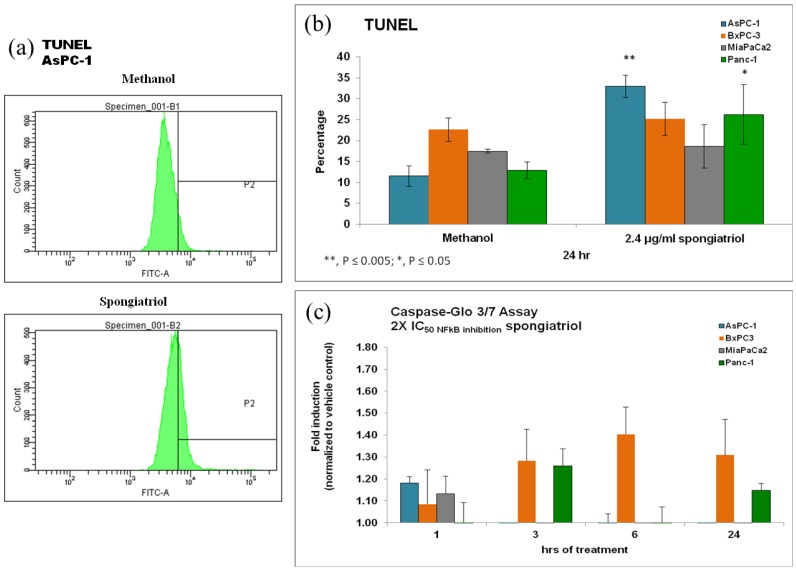
(**a**) Flow cytometry histograms for TUNEL in AsPC-1 cells treated for 6 h with vehicle control or 6.8 μM (2.4 μg/mL) spongiatriol (2× IC_50_ for NFκB inhibition in the reporter cell line). One representative experiment is shown. (**b**) Graphical representations of the flow cytometry data showing the average of 3 experiments. Error bars represent standard deviation. Statistical significance was determined through the Student’s *t*-test. (**c**) Graphical representations of the caspase cleavage data showing the average of three experiments. Error bars represent standard error of the mean.

To ascertain if the cytotoxicity observed was caused by apoptosis, terminal deoxynucleotidyl transferase dUTP nick end labeling (TUNEL) was used to detect DNA fragmentation via flow cytometry. In addition, cleavage of caspase 3/7 was also monitored using the commercially available *Caspase-Glo* assay. As shown in Figure 4, spongiatriol induced modest but statistically significant apoptosis in the AsPC-1 and Panc-1 cell lines. Both of these cell lines are known to have constitutively activated NFκB [9]. Moderate caspase 3/7 cleavage was seen in AsPC-1, BxPC-3 and MiaPaCa-2 within 1 h of treatment and still moderate but slightly more cleavage in BxPC-3 cells at 3, 6 and 24 h of treatment and in Panc-1 cells at 3 and 24 h of treatment. Basal caspase 3 cleavage has been reported in 3 of the 4 pancreatic cancer cell lines used [17] and this basal expression may have masked any further induction of caspase cleavage. As for the discrepancy between caspase cleavage and TUNEL, it may be due to the timing of measurement since maximum caspase cleavage for BxPC-3 cells was seen at 6 h while TUNEL was measured at 24 h.

**Figure 5:**
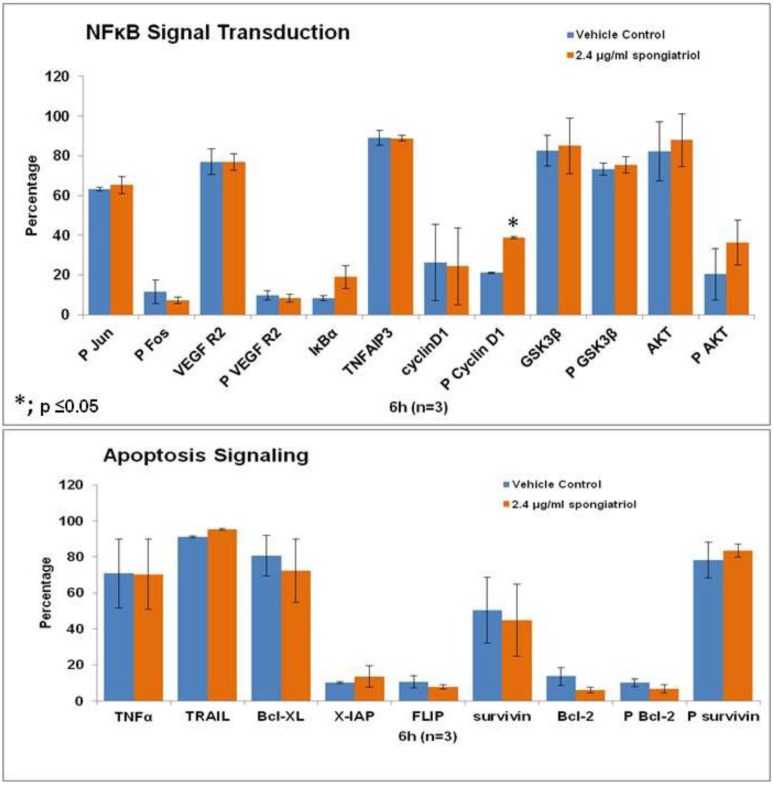
Flow cytometry data for differential protein expression of key components of NFκB and apoptosis signaling performed in AsPC-1 cells treated for 6 h with vehicle control or 2.4 μg/mL spongiatriol. The graphs show the average of three experiments. Error bars represent standard error of the mean. Statistical significance was determined through the Student’s *t*-test.

Although many small molecules that inhibit NFκB have been identified, none are currently used in the clinic, perhaps because their mode of action is not specific to this pathway [15]. The small molecule nimbolide has recently been shown to inhibit NFκB by interacting with the IκB kinase (IKK) [18] and is a promising potential therapeutic. Therefore, initial studies to understand the effects of spongiatriol treatment on downstream signaling pathways in pancreatic cancer cells were undertaken. The AsPC-1 cell line was chosen for these experiments as it has constitutively active NFκB and is the most metastatic and resistant to apoptosis of the four cell lines used in these studies. Differential protein expression in AsPC-1 cells treated with vehicle control or 6.8 μM (2.4 μg/mL) spongiatriol for 6 h was conducted for selected proteins involved in both NFκB and apoptotic signaling using flow cytometry. The proteins selected are all regulated by NFκB and are associated with either cell survival (Bcl-2, phosphorylated Bcl-2 (Ser70; P Bcl-2), Bcl-xL, X-IAP, phosphorylated survivin (Thr34; P surviving)); induction of apoptosis (TNFα, TRAIL); proliferation (cyclin D1, phosphorylated cyclin D1 (Thr 286; P Cyclin D1), P survivin); or angiogenesis (VEGF Receptor 2, phosphorylated VEGF R2 (Tyr 1059; P VEGF R2)). In addition, we looked at proteins known to inhibit NFκB (IκBα, TNFAIP3, GSK3β, phosphorylated GSK3β (Ser9; P GSK3β)) and proteins upstream of NFκB (phosphorylated Fos (Ser 32; P Fos), phosphorylated Jun (Ser73; P Jun), Akt, phosphorylated Akt (Ser 473; P Akt)). The results from 3 experiments were compared using a student’s *t*-test (Figure 5). Of the proteins investigated, only the increase in levels of phosphorylated cyclin D1 caused by spongiatriol treatment was statistically significant. Phosphorylation of cyclin D1 targets it for degradation and an increase in P-cyclin D1 was expected upon inhibition of NFκB and should lead to a block in cell proliferation. The differences in IκBα and phosphorylated Akt were close to being significant. Overall interesting trends were observed, but the lack of statistically significant differences versus control suggests that the proteins selected for this study are not of major importance to the mode of action of spongiatriol at the six hour time point. These experiments were not exhaustive and further experiments must be performed to better understand the cellular effects of spongiatriol caused by inhibition of NFκB.

## 3. Experimental Section

### 3.1. Reagents

Compounds were obtained from the Harbor Branch Oceanographic Institute at Florida Atlantic University pure natural product compound library. Spongiatriol stock solution was used at a concentration of 1 mg/mL in methanol. Methanol and isopropanol used in the experiments were purchased from Fisher Scientific, Fair Lawn, NJ, USA. The 3-(4,5-dimethyl-2-thiazolyl)-2,5-diphenyl-2*H*-tetrazolium bromide (MTT) used for cell viability assays was purchased from Sigma Chemical Co., St. Louis, MO, USA.

### 3.2. Cell Culture

The human pancreatic cancer cell lines PANC-1 (CRL-1469), AsPC-1 (CRL-1682), BxPC-3 (CRL-1687) and MIA PaCa-2 (CRL-1420) cell lines were obtained from ATCC (Manassas, VA, USA), grown, aliquotted and maintained in liquid nitrogen. Aliquots of AsPC-1, Panc-1, and BxPC-3 were thawed and grown in RPMI-1640 (ATCC, Manassas, VA, USA) supplemented with 10% Fetal Bovine Serum (Hyclone, Logan, UT, USA), 0.11 mg/mL Sodium Pyruvate, 4.5 g/L d-glucose, 18 mM HEPES Buffer, 100 U/mL penicillin G sodium, 100 μg/mL streptomycin sulfate, 0.25 μg/mL amphotericin B, 2 mM l-glutamine and 50 μg/mL gentamicin (Complete RPMI). Sodium pyruvate and glucose were purchased from Sigma, St Louis, MO, USA. All other supplements were purchased from Gibco, Carlsbad, CA, USA. The MIA PaCa-2 cell line was grown in DMEM media (ATCC, Manassas, VA, USA) supplemented with 5% horse sera (ATCC, Manassas, VA, USA), 10% Fetal Bovine Serum (Hyclone, Logan, UT, USA), 100 U/mL penicillin G sodium, 100 μg/mL streptomycin sulfate, 0.25 μg/mL amphotericin B and 50 μg/mL gentamicin. Cells were maintained in a humidified incubator at 37 °C and 5% CO2.

### 3.3. NFκB Inhibition (A549/NFκB-luc) Screening Assay

The A549 NFκB-luc cell line was obtained from Panomics (RC0002; Freemont, CA, USA) and is an A549 lung carcinoma cell line stably transfected with a luciferase reporter under the transcriptional control of NFκB. Cells were received at passage 3 from Panomics and were propagated and maintained in complete media containing Dulbecco’s Modified Eagles Medium (ATCC, Mannassas, VA, USA), 10% Fetal Bovine Serum, 100 units/mL penicillin, 100 μg/mL Streptomycin Penicillin, and 100 μg/mL hygromycin B. Cells were plated at a concentration of 5 × 10^4^ cells per well on a clear 96-well plate, allowed to adhere and treated with media containing 100 ng/mL TNF-α (Alexis Biochemicals, San Diego, CA, USA) and either 5 μg/mL of HBOI compounds or vehicle control and incubated for 6 h. Media was removed by aspiration and cells were lysed with detergent lysis buffer (Promega, Madison, WI, USA). Cell solutions were mixed before transferring 20 μL of each lysate to a white 96-well plate. An equal volume of Luciferase substrate (Promega P/N E1500) was added to each well, mixed, and the light produced read immediately using the luminescence setting on a BMG NOVOstar plate reader. To determine cell viability, 20 μL of each lysate were also plated on a clear-bottom black 96-well plated and equilibrated with ethidium bromide for 30 min. The resulting fluorescence was read on the fluorescence setting of the BMG NOVOstar plate reader. A compound was considered a hit if it showed 50% inhibition of light production with less than 20% cytotoxicity. To determine the concentration of spongiatriol necessary to produce 50% inhibition (IC_50_), serial dilutions ranging from 0.00013 to 5 μg/mL were used in the assay described above. The resulting absorbencies were normalized against methanol treated cells using Microsoft Excel, and this data was subjected to a non-linear regression curve fit with GraphPad Prism 5 software (La Jolla, CA, USA).

### 3.4. Cell Viability Assay (MTT)

Twelve thousands cells were plated on a 96-well tissue culture plate. Cells were allowed to adhere for 24 h. At the end of this incubation, 100 μL of medium were removed from each test well and 100 μL of medium containing treatment were added. Treatment consisted of a range of concentrations from 0.00013 to 5 μg/mL of spongiatriol or media with methanol. The cells were then incubated for 72 h at 37 °C and 5% CO_2_. After this incubation, 75 μL 5 mg/mL 3-(4,5-dimethylthiazol-2-yl)-2,5-diphenyltetrazolium bromide (MTT) were added to each well. The cells were then incubated for 3 h at 37 °C. The plates were centrifuged for 10 min at 800 rpm. The supernatant was removed and 200 μL acidified isopropyl alcohol (1:500 solution of hydrochloric acid to isopropanol) were added to each well. The plates were shaken for 15 min. The absorbencies of these solutions were measured at 570 nm with a plate reader (NOVOstar, BMG Labtech Inc., Durham, NC, USA). The resulting absorbencies were normalized against methanol treated cells using Microsoft Excel. Determination of the dose at which 50% inhibition is found (IC_50_) was done using a non-linear regression curve fit with GraphPad Prism 5 software. 

### 3.5. Caspase-Glo

To determine induction of apoptosis, a commercially available kit from Promega (G0890; Madison, WI, USA) was used to measure cleavage of caspase 3/7 following manufacturer’s protocol. Briefly, 10,000 cells were plated in a 96-well flat bottom white plate. Cells were allowed to adhere overnight then treated with 2.4 μg/mL spongiatriol (2× IC_50_ for NFκB inhibition in the reporter cell line) or its vehicle control for 1, 3, 6 or 24 h. At the end of treatment, an equal volume of pro-luminescent caspase 3–7 substrate was added to the wells and incubated at room temperature with mild shaking for 30 min protected from light. In cells with active caspase 3/7, the tetrapeptide sequence DEVD is cleaved to release aminoluciferin, and the resulting luminescence was read with a plate reader (NOVOstar, BMG Labtech Inc., Durham, NC, USA). Luminescence results are presented as fold induction compared to the vehicle control treated sample.

### 3.6. TUNEL

To determine induction of apoptosis, a commercially available kit from Promega (G3250; Madison, WI, USA) to measure fragmented DNA by incorporating fluorescein 12-dUTP at 3′-OH DNA ends using the Terminal Deoxynucleotidyl Transferase enzyme was used following manufacturer’s protocol. Briefly, 500,000 cells were plated in a 6-well plate. Cells were allowed to adhere overnight then treated with 2.4 μg/mL spongiatriol (2× IC_50_ for NFκB inhibition in the reporter cell line) or its vehicle control for 24 h. At the end of treatment, cells were trypsinized, fixed with 4% paraformaldehyde and permeabilized with ice cold ethanol. Cells were transferred to a 96 well U-bottom plate, equilibrated for five minutes and then treated with 25 μL of a reaction mix containing the nucleotide mix and the enzyme to label the fragmented DNA for 60 min at 37 °C. The reaction was stopped by the addition of 20 mM EDTA. The resulting fluorescence was visualized via flow cytometry in a BD FACSCanto equipped with a plate adapter, acquiring 20,000 events.

### 3.7. Intracellular Staining

To determine the levels of particular signal transduction molecules affected by spongiatriol, AsPC-1 cells were treated for six hours with 2.4 μg/mL spongiatriol or its vehicle control (methanol). At the end of treatment, cells were trypsinized, fixed with 4% paraformaldehyde and permeabilized with ice cold ethanol. Cells were labeled with primary antibodies specific for the active (phosphorylated) form of NFκB using a phycoerythrin conjugated antibody (PE Mouse anti-NF-κB p65 (pS529); 558423; BD Pharmingen, San Diego, CA, USA) or a mouse IgG isotype (Jackson ImmunoResearch, West Grove, PA, USA) for 1 h at room temperature. For differential protein expression studies cells were labeled with unconjugated antibodies against phosphorylated survivin Thr34 (NF500-236, Novus Biologicals, Littleton, CO, USA), Bcl-2 (4223S), phosphorylated Bcl-2 Ser70 (2827S), Bcl-xL (2764), X-IAP (2045), TNFα (6945S), TRAIL (3219S), cyclin D1(2978), phosphorylated cyclin D1 Thr 286 (2921S), VEGF Receptor 2 (2479S), phosphorylated VEGF R2 Tyr 1059 (3817S), IκBα (2682P), TNFAIP3 (5630S), GSK3β (9315), phosphorylated GSK3β Ser9 (9323), phosphorylated Fos Ser 32 (5348P), phosphorylated Jun Ser73 (3270P), Akt (9272), phosphorylated Akt Ser 473 (9271S) (Cell Signaling Technologies, Danvers, MA, USA) or a rabbit IgG isotype (Jackson ImmunoResearch, West Grove, PA, USA) for 1 h at room temperature. Cells were labeled with a secondary phycoerythrin conjugated secondary antibody (Jackson ImmunoResearch, West Grove, PA, USA) for all antibodies except NFkB for 30 min at room temperature. Cells were washed and resuspended in 2% FBS in PBS and analyzed via flow cytometry in a BD FACSCanto equipped with a plate adapter, acquiring 20,000 events.

## 4. Conclusions

Spongiatriol is a marine natural product known to reduce blood pressure in rats [19] and to induce apoptosis and cell cycle arrest in MM96E melanoma cells [20]. The effects of spongiatriol on pancreatic cancer cells were unknown. We show here that spongiatriol can significantly reduce NFκB transcriptional activity in the A549 NFκB-luc reporter cell line and significantly reduce the levels of phosphorylated NFκB present in the AsPC-1 pancreatic adenocarcinoma cell line. In addition, spongiatriol significantly, albeit moderately, induced apoptosis both in the AsPC-1 and the Panc-1 cell lines and showed cytotoxicity in the low macromolar range in the AsPC-1, Panc-1 BxPC-3 and MiaPaCa2 cell lines.

It remains to be ascertained whether the inhibition of NFκB by spongiatriol leads to functional effects such as reduced inflammation or angiogenesis that would make it a potential therapeutic useful against pancreatic cancer. Furthermore, its full mode of action and molecular target remain to be identified. However, the ability to produce the compound through synthesis [21] along with the results reported here suggests that spongiatriol may merit further study.
